# Two Species of Long-Day Breeding Hamsters Exhibit Distinct Gut Microbial Responses to Photoperiodic Variations

**DOI:** 10.3390/ani15111648

**Published:** 2025-06-03

**Authors:** Chao Fan, Huiliang Xue, Jinhui Xu, Ming Wu, Lei Chen, Laixiang Xu

**Affiliations:** School of Life Sciences, Qufu Normal University, Qufu 273165, China

**Keywords:** gut microbiota, rodents, photoperiods, species specific, 16S rRNA gene

## Abstract

The relationship between the gut microbiota and photoperiod has received widespread attention, and it is necessary to explore the probable common mechanisms involved. We tested whether the gut microbiota of animals with similar light-regulated life history traits would also exhibit consistent responses to the photoperiod. Here, two species of long-day breeders, striped hamsters (*Cricetulus barabensis*) and Djungarian hamsters (*Phodopus sungorus*), were raised under different photoperiods (long daylight, LD; short daylight, SD), and their cecal contents were collected to assess the gut microbiota. There was no difference in the gut microbial diversity between the groups of striped hamsters; however, in the Djungarian hamsters, lower Chao and Shannon indices were observed in the LD group than in the SD group. The bacterial community variation in the striped hamsters was reflected mainly in the enrichment of the genera *Enterorhabdus* and *Jeotgalicoccus* in the LD group; meanwhile, more taxa with significant changes in relative abundance under different photoperiods were found in the Djungarian hamsters, such as the enrichment of the genera *Lactobacillus* and *Faecalibaculum* in the LD group and the enrichment of the genera *Ruminococcus* and *Colidextribacter* in the SD group. The LD conditions substantially reduced the complexity of the gut microbial network in the Djungarian hamsters and increased the *R*^2^ value of the striped hamster gut microbiota under fitting with a neutral community model. Moreover, the potential gut microbial functions in the striped hamsters were relatively stable, but variations were observed in multiple pathways between the groups of Djungarian hamsters. These results contribute to the understanding of host species specificity in the response of the gut microbiota to external changes.

## Simple Summary

The regulatory role of photoperiod on animals is crucial, and its connection with the gut microbial communities is now receiving increasing attention. The aim of this study was to investigate whether the gut microbiota of animals with similar light-regulated life history traits exhibit consistent responses to the photoperiod. Through laboratory lighting control experiments, we found that two species of long-day breeding rodents, striped hamsters (*Cricetulus barabensis*) and Djungarian hamsters (*Phodopus sungorus*), exhibited distinct gut microbial responses in diversity, bacterial composition and potential functional features to the photoperiodic variations. This further implies that there may be distinct variations in the role played by the gut microbiota of different animals in assisting the host in adapting to environmental changes.

## 1. Introduction

The gut microbiota is a complex and dynamic ecosystem composed of trillions of microorganisms that play pivotal roles in maintaining host health and is involved in numerous physiological processes, such as digestion, reproduction, immune regulation, and even neurological functions [1,2,3]. Thus, understanding the factors and corresponding mechanisms that influence the composition and function of the gut microbiota is essential for zoological and ecological research. In recent years, research has increasingly highlighted the profound connections between the gut microbiota and external environmental factors, such as diet, geographical location, and climate [4,5]. These variables typically cause changes in the gut microbiota that can effectively help hosts adapt to the dynamic living environment. For example, a high-protein diet not only directly affects the gut microbiota of rat dams (*Rattus norvegicus*) but also indirectly affects that of their offspring by altering the composition of the maternal milk, which results in an increase in the abundance of various probiotics, ensuring the healthy growth of the young rats [6]. The gut microbiota of wild plateau pikas (*Ochotona curzoniae*) exhibits clear altitude-associated distributions, and different bacterial community types are associated with increased fitness of the host [5,7]. Among the environmental variables, some exhibit periodic changes that often lead to regular fluctuations in animal physiology and the gut microbiota, and photoperiod is one such variable.

Photoperiod refers to the durations of light and dark periods within a day and is an important external factor influencing a majority of mammalian rhythms [8]. Variations in light duration can lead to significant changes in animal physiology, behavior, and redox equilibrium, and participates in regulating the seasonal reproductive activities of animals [8,9,10]. In recent years, the relationship between photoperiod and the gut microbiota has gradually become a research hotspot. Researchers have reported that different photoperiods can affect specific bacteria, leading to variations in the functional characteristics of the gut microbiota in photoperiod-sensitive animals, such as birds and small mammals [11,12]. Photoperiod manipulation had a significant impact on the gut microbial composition of chickens (*Gallus gallus domesticus*) and caused the differential expression of genes related to the biological clock [13]. Continuous darkness resulted in the disappearance of rhythmic oscillations in nearly all intestinal regions of mice (*Mus musculus*), which was accompanied by changes in the relative abundance of Clostridia [14]. Melatonin, regulated by photoperiod, can improve intestinal homeostasis in Mongolian gerbils (*Meriones unguiculatus*) by altering the composition of gut microbiota and reducing inflammation [12]. In Fisher 344 rats, increasing the daylight exposure time altered the gut microbiota, which was closely associated with host weight gain and fat parameters [15]. In addition, short periods of daylight exacerbated the negative effects of antibiotics on the gut microbiota in mice, inducing an imbalance in the purine metabolism pathway [16]. The main purpose of these studies was to explore whether the light duration control measures widely used in the fields of husbandry and medicine might cause gut ecological dysbiosis and to make reasonable adjustments to light/dark allocation to ensure high productivity in the breeding industry or the effectiveness of medical treatments. There are currently few reports exploring the shared or divergent characteristics of gut microbial responses to photoperiods among different animal species.

This study aims to explore whether the gut microbiota of animals with similar light-regulated life history traits exhibit analogous responses to the photoperiod. Small rodents have a significant impact on both natural and artificial ecosystems, and most of those living in the Northern Hemisphere are long-day breeders; thus, population outbreaks of these rodents have clear seasonal characteristics. Owing to their significant impacts on agriculture and animal husbandry, these animals have attracted the attention of researchers and rodent pest control personnel, increasing the interest in the “photoperiod–gut microbiota–host” regulatory network. In particular, the gut microbiota is associated with the effect of photoperiod on seasonal breeding [17]. The striped hamster (*Cricetulus barabensis*) and Djungarian hamster (*Phodopus sungorus*) are two important species of long-day breeding rodents; both belong to the family Cricetidae and are excellent experimental subjects for studying seasonal reproduction [18,19]. Previous studies on the effects of photoperiod on striped hamsters have focused mainly on the physiological level of individuals and have not yet addressed the symbiotic gut microbiota [18,20,21]. Studies exploring the connections between the gut microbiota and photoperiod in Djungarian hamsters have focused on certain specific bacteria and their functions in physiological and behavioral regulation, while a comprehensive assessment of the internal microbial community is lacking [22,23,24]. Therefore, we selected these two hamster species as the research subjects. The variations in the gut microbiota among individuals under long and short photoperiods were compared through a rigorous controlled experiment, and the inconsistency of these variations among different hamster species was also explored. The results confirm the host species specificity of the photoperiodic response of the gut microbiota, further implying that there may be clear variations in the role played by the gut microbiota of different animals in assisting the host in adapting to environmental changes.

## 2. Materials and Methods

### 2.1. Experimental Design and Sample Collection

The striped hamsters and Djungarian hamsters were bred in our colony maintained at Qufu Normal University, avoiding the residual influence of the primitive environmental background of the wild populations. Each hamster was housed separately in an opaque plastic chamber to prevent bacterial transfer between them and was placed in a light controller with an adaptive treatment photoperiod of 12L:12D at 22 ± 2 °C and a relative humidity of 55 ± 5%. Then, twelve adult male individuals of each hamster species were randomly divided into two groups, with half assigned to the LD group (long daylight, light/darkness = 16 h:8 h) and the other half to the SD group (short daylight, light/darkness = 8 h:16 h). Water and the same artificial rodent feed (Qianmin Feed, Shenyang, Liaoning, China) (Table S1) were provided ad libitum to all the rodents, and all other conditions were the same except for the photoperiod. After four weeks, all hamsters were killed by CO_2_ asphyxiation, the cecum was removed, and the contents were flash frozen in 2 mL cryogenic vials (Corning, Reynosa, Tamaulipas State, Mexico) and stored in a −80 °C ultralow-temperature freezer (Thermo Fisher, Waltham, MA, USA).

All procedures followed the Laboratory Animal Guidelines for the Ethical Review of Animal Welfare (GB/T 35892-2018) [25] and were approved by the Biomedical Ethics Committee of Qufu Normal University (permit number: 2022059).

### 2.2. DNA Extraction and Sequencing

Genomic DNA was extracted from the cecal contents via MJ DNA Kits according to general protocols (Majorbio, Shanghai, China). All the DNA samples were subjected to quality control, and the concentration was quantified with a NanoDrop 2000 spectrophotometer (Thermo Fisher, Wilmington, DE, USA). The bacterial 16S rDNA fragments (V3–V4 regions) were amplified from the extracted DNA using the primers 338F (5′-ACTCCTACGGGAGGCAGCAG-3′)/806R (5′-GGACTACHVGGGTWTCTAAT-3′), and the following polymerase chain reaction (PCR) conditions were used: 30 s at 95 °C, followed by 27 cycles of 30 s at 55 °C and 45 s at 72 °C. PCRs were performed with 4 μL of 5 × TransStart FastPfu buffer, 2 μL of 2.5 mM deoxynucleoside triphosphates (dNTPs), 0.8 μL of each primer (5 μM), 0.4 μL of TransStart FastPfu DNA Polymerase, 10 ng of extracted DNA, and ddH_2_O to bring the total volume up to 20 μL. Agarose gel electrophoresis was performed to verify the size of the amplicons. Amplicons were subjected to paired-end sequencing on the Illumina MiSeq platform using PE300 chemicals at Majorbio Bio-Pharm Technology Co. Ltd. (Shanghai, China). The raw reads were deposited into the National Center for Biotechnology Information database under accession number PRJNA1166740.

### 2.3. Bioinformatics and Statistical Analysis

The sequences obtained after demultiplexing were merged using FLASH (v1.2.11) and quality filtered with fastp (v0.19.6) [26,27]. Then, they were denoised using the Divisive Amplicon Denoising Algorithm 2 (DADA2) plugin in QIIME2 (v2020.2) software by filtering out noisy, chimeric, and singleton sequences and correcting errors in edge sequences [28], obtaining the DADA2-denoised sequences called amplicon sequence variants (ASVs). The taxonomic assignment of ASVs was conducted by the naive Bayes consensus taxonomy classifier integrated in the QIIME2 pipeline according to the SILVA bacterial 16S rRNA database (v138). ASVs with a relative abundance less than 0.001 in at least 23 samples or belonging to chloroplasts and mitochondria were removed before performing downstream analysis. To reduce the impact of varying sequencing depths, normalization was conducted based on the smallest sequence count among all samples, and every sample was rarefied to 24,830 sequences, still achieving a coverage index of more than 99.9%. Alpha diversity indices (Chao and Shannon indices) calculated by Mothur (v1.30.2) were employed to assess intestinal microbial richness and diversity, while beta diversity distance matrices were generated using the QIIME2 computational platform. The functional profiles of bacteria were inferred using PICRUSt2 software (v2.2.0-b) with reference to the Kyoto Encyclopedia of Genes and Genomes (KEGG) database. The functional pathway abundance tables across three distinct levels were generated by aligning KOs (KEGG Orthology) against the KEGG database [29].

Statistical analysis was mainly performed using the Majorbio Cloud platform (https://cloud.majorbio.com/) and R software (v3.5.3). The Wilcoxon rank-sum test was applied to detect differences in alpha diversity indices. Principal coordinate analysis (PCoA) and Adonis analysis (PERMANOVA, permutational multivariate analysis of variance) were conducted by using the R packages “vegan (v2.5.3)” and “ggplot2 (v3.1.0)”. The heatmap was generated by the R package “pheatmap (v1.0.10)”, and linear discriminant analysis effect size (LEfSe) was used to identify the microbial taxa that contributed the most to the differences between groups. Significant pairwise correlations among ASVs were identified by selecting Spearman’s correlations with *p*-values less than 0.05 and absolute coefficient values exceeding 0.6, followed by the use of Gephi software (v0.9.2) to construct networks. An UpSet diagram was used to display the distribution of ASVs among the different experimental groups. To determine the potential importance of stochastic processes for gut microbial community assembly, a neutral community model (NCM) was used to predict the relationship between ASVs detection frequency and mean relative abundance. Functional differences within KEGG pathways were detected and illustrated using the STAMP v2.1.3 software.

## 3. Results

### 3.1. Effects of Photoperiods on the Gut Microbial Diversity of the Two Hamster Species

**Figure 1:**
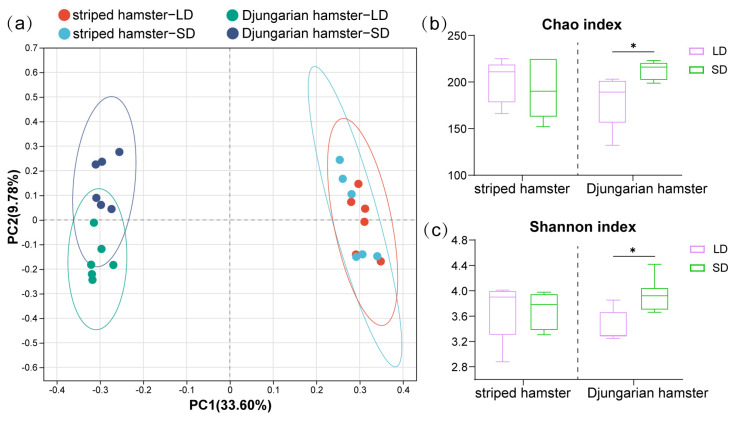
Gut microbial diversity of two hamster species under different photoperiods. (**a**) Principal coordinate analysis (PCoA) based on Bray–Curtis distances calculated using ASVs; (**b**,**c**) Chao and Shannon indices of the two hamster species. Differences between the LD and SD groups were assessed by Wilcoxon rank-sum tests and are denoted as * *p* < 0.05.

After data processing, we obtained 595,920 high-quality gene sequences of bacterial 16S rRNA from all the samples, and they were clustered into 550 ASVs. The rarefaction curves of the Sobs index revealed that the number of ASVs finally stabilized when the number of sampled reads increased, and there was no further fluctuation or growth (Figure S1). After performing PCoA on the basis of the Bray-Curtis dissimilarities of the ASVs (Figure 1a), we found that the LD and SD groups of striped hamsters were not separated from each other (*R*^2^ = 0.099, *p* = 0.346), whereas the sample positions representing the different light treatment groups of Djungarian hamsters were significantly separated (*R*^2^ = 0.228, *p* = 0.003). Under the different lighting conditions (Figure 1b,c), there was no significant change in the Chao (*p* = 0.573) or Shannon (*p* = 0.575) indices of the gut microbiota in striped hamsters. However, the LD group had a lower Chao index (*p* = 0.013) and a lower Shannon index (*p* = 0.013) than the SD group of Djungarian hamsters.

### 3.2. Alterations in the Gut Microbial Composition of the Two Hamster Species

**Figure 2:**
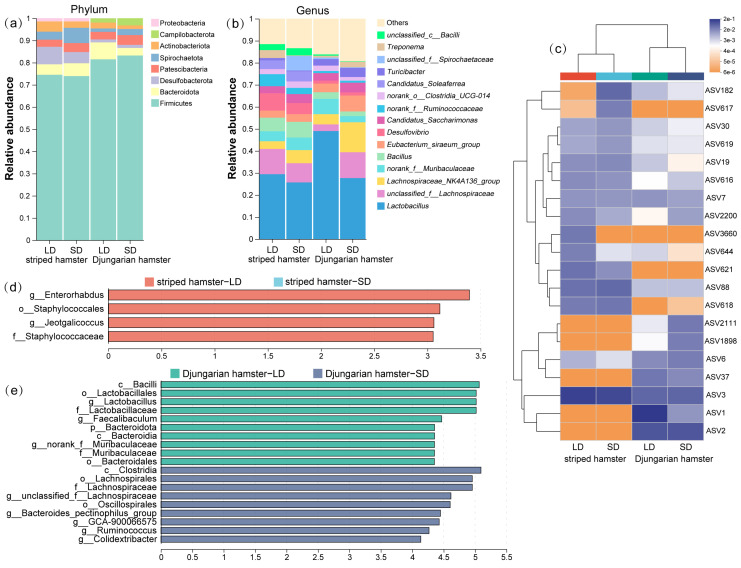
Variations in the gut microbial composition resulting from photoperiods. (**a**,**b**) Taxonomic compositions at the phylum and genus levels; (**c**) cluster heatmap drawn using the 20 most abundant ASVs; (**d**,**e**) LEfSe identification of gut microbial taxa with significant differences between the LD and SD groups (LDA > 3, *p* < 0.05).

We explored the distribution of numerically abundant bacterial taxa in each group and found that the gut microbial communities of striped hamsters and Djungarian hamsters presented a certain degree of similarity at both higher and lower classification levels (Figure 2a,b): Firmicutes, Bacteroidota, and Desulfobacterota were the major phyla; and *Lactobacillus*, *unclassified_f__Lachnospiraceae,* and *Lachnospiraceae_NK4A136_group* were the most abundant components at the genus level. However, marked variations in the gut bacteria of the two hamster species under different photoperiod conditions were observed. Among the top 20 abundant ASVs, greater changes were observed in Djungarian hamsters; the heatmap displayed more color blocks with varying hues, and the distribution of ASV blocks with color differences was also inconsistent between the two experimental species (Figure 2c). We performed LEfSe on the taxa that presented LDA scores greater than 3.0 to identify the bacteria that presented the greatest variations under different lighting conditions (Figure 2d,e). In striped hamsters, the genera *Enterorhabdus* and *Jeotgalicoccus* were significantly enriched in the LD group. In Djungarian hamsters, the relative abundances of many gut microbial taxa, such as the genera *Lactobacillus* and *Faecalibaculum*, were much greater in the LD group, whereas the family Lachnospiraceae and genus *Ruminococcus* were more abundant in the SD group.

### 3.3. Differences in Gut Microbial Networks Induced by Photoperiods

**Figure 3:**
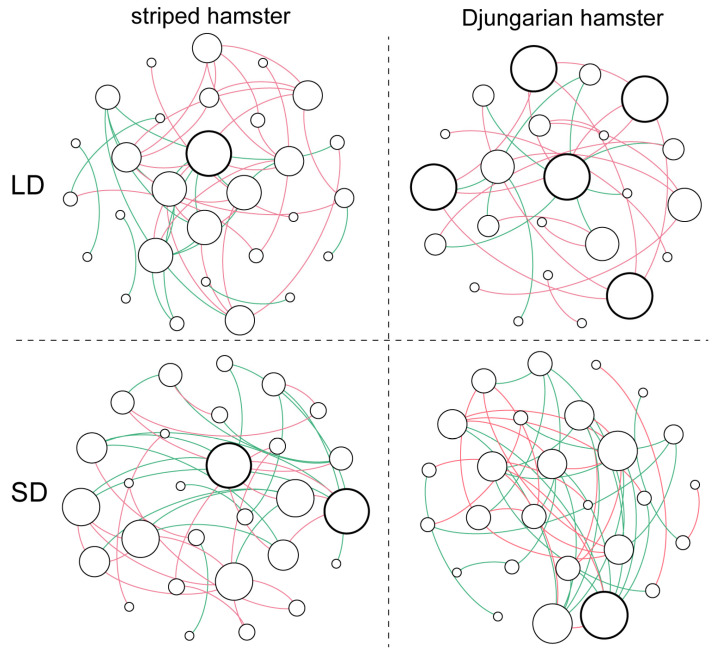
Co-occurrence networks of the top 30 abundant ASVs. Each node represents an ASV, and its size indicates the degree. Bold circles represent the nodes with the highest degree. Links represent significant (*p* < 0.05) and strong (Spearman’s correlation greater than 0.6 or lower than −0.6) correlations (red: positive; green: negative).

By using the top 30 abundant ASVs to construct each group’s co-occurrence network (Figure 3, Table S2), we found that the gut microbiota of the striped hamsters in the LD group had a slightly more complex network structure than that of the striped hamsters in the SD group, especially in terms of the number of total triangles (LD: 28 vs. SD: 17). However, the gut microbial network of the LD Djungarian hamsters was much less complex than that of the SD Djungarian hamsters, as reflected in various topological properties, such as total links (LD: 25 vs. SD: 45) and average degree (LD: 2.174 vs. SD: 3.333). Notably, the differences in photoperiod also altered the pattern of interactions among the gut microbes. In both hamster species, long daylight increased the proportion of positive links between the major bacteria (striped hamster, LD: 59.1% vs. SD: 55.0%; Djungarian hamster, LD: 72.0% vs. SD: 44.4%). Additionally, the node that had the highest degree in the LD group of the striped hamsters represented an ASV belonging to the genus *Desulfovibrio*; however, in the SD group, two ASVs belonging to the genus *Lactobacillus* became hubs of link aggregation. Among the Djungarian hamsters, five ASVs with inconsistent taxonomies were the main nodes that occurred in the gut microbial network of the LD group, whereas an ASV belonging to the family Oscillospiraceae was the node with the highest degree in the SD group.

### 3.4. Distribution and Assembly Processes of Gut Microbial Communities

**Figure 4:**
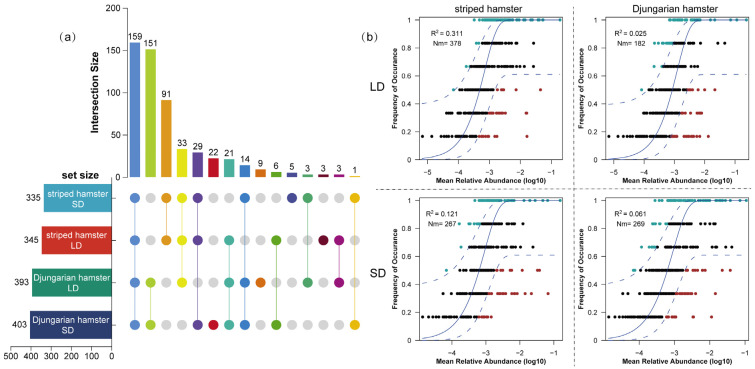
Distribution and assembly processes of gut microbial communities in two species of hamsters. (**a**) UpSet diagram of the ASV distribution among groups; (**b**) fit of the neutral community model: solid blue lines indicate the best fit to the model, whereas dashed blue lines represent 95% confidence intervals around the prediction.

As relatively closely related species within the family Cricetidae, the striped hamsters and Djungarian hamsters exhibited a substantial overlap in their gut microbiota, with 159 ASVs shared among all the groups (Figure 4a). The number of shared ASVs between the two light-treated groups of Djungarian hamsters and the corresponding groups of striped hamsters were 151 and 91, respectively. However, the number of ASVs unique to each group was relatively small, suggesting that the effect of photoperiods on foundational gut bacteria is not reconstructive. The NCM reflects the relationship between the relative abundance and occurrence frequency of ASVs, and the parameter *R*^2^ represents the overall fit of this model, whereas *Nm* is an estimate of dispersal between communities (Figure 4b). The *R*^2^ value of all groups was lower than 0.5, indicating the dominance of deterministic processes in the gut microbial community assembly of captive animals. The *R*^2^ and *Nm* values in the LD striped hamsters (0.311; 378) were both greater than those in the SD striped hamsters (0.121; 267), suggesting that long daylight periods increased the influence of stochastic processes on community assembly. However, for the Djungarian hamsters, long daylight weakened the influence of stochastic processes, as the *R*^2^ and *Nm* values decreased from 0.061 to 0.025 and from 269 to 182, respectively.

### 3.5. Differences in Gut Microbial Functions

**Figure 5:**
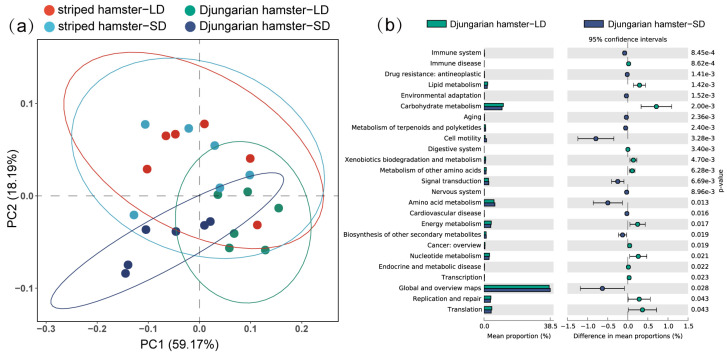
Differences in gut microbial functions. (**a**) Principal coordinate analysis (PCoA) based on Bray–Curtis distances of KOs; (**b**) significant differences in level 2 KEGG pathways between different groups of Djungarian hamsters.

Through PCoA based on the Bray–Curtis distance of the KOs (Figure 5a), we found that there was no significant separation between the striped hamster individuals in the LD and SD groups (*R*^2^ = 0.024, *p* = 0.872), whereas functional aspects of the intestinal microbiota in the Djungarian hamsters were altered due to the effects of photoperiod (*R*^2^ = 0.530, *p* = 0.003). By using STAMP to verify the significant difference in level 2 KEGG pathways (Figure 5b), we found that the genes involved in pathways such as lipid metabolism, carbohydrate metabolism, and translation were enriched in the gut microbiota of the LD Djungarian hamsters, whereas the genes involved in the pathways of cell motility, amino acid metabolism, and global and overview maps were enriched in the SD Djungarian hamsters.

## 4. Discussion

Changes in alpha diversity can intuitively reflect the patterns of the gut microbial response to photoperiod. A light manipulation experiment on Brandt’s voles (*Lasiopodomys brandtii*) demonstrated that neither the long-day group (16 h:8 h) nor the short-day group (8 h:16 h) exhibited significant differences in alpha diversity during or after treatment [17]. Another study also revealed no differences in the gut microbial alpha diversity in Fisher 344 rats between the group exposed to 18 h and that exposed to 6 h of light daily [15]. This is similar to the results for the striped hamsters in our study, suggesting that the influence of photoperiod on gut bacterial diversity in small mammals is generally not very pronounced. However, the gut microbial diversity of the Djungarian hamsters exhibited a more sensitive response to photoperiod changes, with both the Chao index and Shannon index under long-day conditions being lower than those under short-day conditions. This aligns with the findings from a previous avian study demonstrating that extended light exposure reduced the microbial community diversity in layer chickens (*Gallus gallus domesticus*) [13], confirming the photosensitivity of gut bacteria in Djungarian hamsters. The intestinal bacteria of lower animals appear to exhibit another light-responsive rhythm; for example, carp (*Cyprinus carpio haematopterus*) under a short photoperiod exhibited a reduced gut microbial diversity [30]. Notably, in these cross-species studies involving beta diversity analysis with dimensionality reduction, samples from different photoperiod treatment groups exhibited a distinct separation [13,15,17,30]. This phenomenon was also observed in the Djungarian hamsters, which indirectly underscores the stability of the gut microbial community in striped hamsters when confronted with photoperiodic variations.

Both the striped hamster and the Djungarian hamster, as members of the Cricetidae family, exhibited considerable similarity in their gut microbiota when maintained under identical laboratory conditions, sharing relatively consistent predominant bacterial groups across higher and lower taxonomic levels. However, the photoperiod-responsive patterns of these bacteria significantly differed between the two experimental animal species. The abundance of the genus *Enterorhabdus* was negatively correlated with the concentration of proinflammatory cytokines, and this genus is considered to play a positive role in protecting the gut, whereas the genus *Jeotgalicoccus* may contribute to maintaining neurological health and treating depression [31,32]. These bacteria presented greater relative abundances in the LD group than in the SD group, indicating that the photoperiodic response of the striped hamster gut microbiota is involved mainly in maintaining intestinal homeostasis. Moreover, in the Djungarian hamsters, the different photoperiods induced changes in the relative abundance of numerous metabolism-related bacterial taxa within the gut microbiota. For example, the class Bacilli and its subordinate genera *Lactobacillus* and *Faecalibaculum* were more abundant in the LD group, whereas the class Clostridia and its subordinate taxa the family Lachnospiraceae and the genus *Ruminococcus* were more abundant in the SD group. *Lactobacillus* is a genus of bacteria capable of fermenting carbohydrates such as glucose to lactic acid [33]. Similarly, *Faecalibaculum* is also associated with carbohydrate metabolism, as a high-sugar diet tends to increase the abundance of this genus in the gut [34]. Therefore, the gut microbiota of the Djungarian hamsters under long-daylight conditions might present greater advantages in carbohydrate metabolism than those under short-daylight conditions. Bacteria of the class Clostridia are involved in protein metabolism, and members of this class, such as the family Lachnospiraceae, are usually potentially beneficial bacterial taxa and the main producers of short-chain fatty acids (SCFAs) [35]. These findings indicated that the gut microbiota of the SD Djungarian hamsters exhibited superior amino acid metabolism capabilities compared with those of the LD group. The aforementioned results were consistent with the conclusions derived from the PICRUSt functional analysis of the gut microbiota: the LD and SD groups of striped hamsters exhibited no distinct separation in distribution in the dimensionality reduction analysis, and it was difficult to identify signature differential microbial functional pathways. In contrast, the Djungarian hamsters presented significant intergroup differences, particularly in certain metabolic pathways. In most cases, a long photoperiod may induce long-day breeding animals to transition into reproductive states, during which individuals often exhibit increased demands for energy intake. This partly explains the observed differences in the composition of the gut microbiota in the Djungarian hamsters. However, striped hamsters may rely less on alterations in the gut microbiota to assist their physiological responses to photoperiodic signaling. Alternatively, it could also be due to the artificial feed providing an excess of nutrients relative to this species’ nutritional requirements.

The complexity of the gut microbial co-occurrence network is closely linked to bacterial interactions within it, and its significant alterations often indicate that the host is subjected to stressors caused by the environment [36,37]. The Djungarian hamsters clearly presented more pronounced changes in the complexity of the gut microbial network structure than that striped hamsters did. In particular, the LD group of Djungarian hamsters presented a much greater proportion of positive correlations than the SD group did, as positive cohesion in the co-occurrence network of the gut microbiota was negatively related to external stress [37]. Notably, changes in the “key pivots” of the microbial community network occurred under different photoperiod conditions. The extended photoperiod led to the formation of centralized network hubs in the gut microbiota of the striped hamsters, whereas it caused the formation of decentralized network hubs in the Djungarian hamsters, suggesting that these two species employ distinct strategies in adapting to environmental changes by modulating microbial interaction patterns [37,38]. From the perspective of the intergroup distribution of ASVs, both the striped hamsters and the Djungarian hamsters presented certain unique ASVs. However, the number of exclusive ASVs under long and short photoperiods was extremely limited, indicating that interspecies differences exert a greater influence on the gut microbiota composition than photoperiods do. When combined with experimental findings from studies without dietary or other conditional controls, the results indicate that among the external factors affecting the animal gut microbiota, the photoperiod likely represents a relatively weak environmental determinant [39,40]. When fitted with an NCM, the extended photoperiod increased the *R*^2^ and *Nm* values for the gut microbiota of the striped hamsters but decreased these parameters for the Djungarian hamsters, indicating that prolonged light exposure might be related to stochastic processes in the gut microbial community assembly of the former species but tended to intensify the deterministic processes governing the latter. In other words, lengthening of the photoperiod acts more like a disturbance or stress factor for the gut microbiome of striped hamsters but can more selectively alter the gut microbial composition in Djungarian hamsters [41].

The striped hamster belongs to the genus *Cricetulus*, while the Djungarian hamster is classified under the genus *Phodopus*. The latter is actually one of the primary sources of pet hamsters, indicating its greater adaptability to artificial environments [42]. In contrast, the striped hamster primarily exists as a wild pest rodent so that strict laboratory-controlled experiments might impose more significant negative stress on them and suppress the sensitivity of their gut microbiota to environmental signals [18,21]. Though the use of a single artificial feed ensures uniformity in dietary conditions, it often leads to the simplification of the gut microbiota in captive animals [43]. Individuals bred in a laboratory lack an internal environment shaped by natural backgrounds, which may exacerbate the aforementioned effects. Given that sampling wild populations introduces more confounding variables, observations conducted under semi-natural conditions with larger sample sizes may yield more effective results. Although the two hamster species in the present study share similar photoperiod-regulated life histories, the native distributions of their wild populations differ. The striped hamster primarily inhabits temperate regions of northern Asia, particularly farmlands in northern China, while the distribution range of the Djungarian hamster extends to higher latitudes encompassing Siberian regions, where there is a greater variation in daylight hours [18,21,23]. After a prolonged coevolutionary process, the gut microbiota of Djungarian hamsters exhibited greater plasticity under photoperiod stress than that of striped hamsters. Nevertheless, this hypothesis, along with its specific mechanisms and associated influencing factors, may require further investigation through methodologies such as gradient photoperiod experiments and experiments with a gradual changing of the photoperiod in future research.

## 5. Conclusions

In summary, in this study, photoperiod manipulation experiments were performed on two common small rodent species in the Northern Hemisphere, the striped hamster and the Djungarian hamster, revealing that their gut microbiota exhibited markedly distinct patterns in response to identical photoperiod changes. Compared with the striped hamster, the Djungarian hamster presented significantly greater variations in gut microbial diversity indices and functional profiles. Furthermore, different bacterial indicators, network structures, and community assembly processes of the intestinal microbiota under long and short photoperiods presented unique characteristics in each species. These results indicate that hosts with similar photoperiod-regulated life histories do not necessarily exhibit analogous gut microbiota responses to photoperiod variations, suggesting that gut microbial communities play diverse roles in facilitating host adaptation to changing environmental conditions.

## Data Availability

The raw reads were deposited into the National Center for Biotechnology Information database under accession number: PRJNA1166740.

## Supplementary Materials

The following supporting information can be downloaded at: https://www.mdpi.com/article/10.3390/ani15111648/s1, Figure S1: Rarefaction curves of each sample; Table S1: Main components of the artificial feed; Table S2: Network indices of gut microbiota in each group.
