# Supplementary material for: Two Species of Long-Day Breeding Hamsters Exhibit Distinct Gut Microbial Responses to Photoperiodic Variations
Source: Animals (Basel). 2025 Jun 3;15(11):1648. doi: 10.3390/ani15111648 (PMC12153784; doi:10.3390/ani15111648)
Supplement: Supplementary file 1 [file animals-15-01648-s001.zip › Table S1.pdf]

**Table S1** Main components of the artificial feed.

| Main materials | Nutrition contents (g/kg) |
|---|---|
| corn, wheat, soybean, animal protein, salt, bone meal, and rice, et al. | Moisture content: ≤100<br>Crude protein: ≥180<br>Crude fat: ≥40<br>Crude fiber: ≤50<br>Crude ash: ≤80<br>Calcium: 10 ~ 18<br>Phosphorus: 6 ~ 12 |
