# Supplementary material for: Two Species of Long-Day Breeding Hamsters Exhibit Distinct Gut Microbial Responses to Photoperiodic Variations
Source: Animals (Basel). 2025 Jun 3;15(11):1648. doi: 10.3390/ani15111648 (PMC12153784; doi:10.3390/ani15111648)
Supplement: Supplementary file 1 [file animals-15-01648-s001.zip › Table S2.pdf]

Table S2 Network indices of gut microbiota in each group.

Network indices	striped hamster		Djungarian hamster	
	LD	SD	LD	SD
Total nodes	29	28	23	27
Total links	44	40	25	45
Positive links	26	22	18	20
Negative links	18	18	7	25
Average Degree	3.034	2.857	2.174	3.333
Network Diameter	6	7	7	6
Average path length	2.288	2.489	3.106	2.062
Density	0.108	0.106	0.099	0.128
Average Clustering Coefficient	0.575	0.505	0.500	0.648
Total triangles	28	17	7	37
