# Supplementary figures and images for: Two Species of Long-Day Breeding Hamsters Exhibit Distinct Gut Microbial Responses to Photoperiodic Variations
Source: Animals (Basel). 2025 Jun 3;15(11):1648. doi: 10.3390/ani15111648 (PMC12153784; doi:10.3390/ani15111648)

## Rarefaction curves

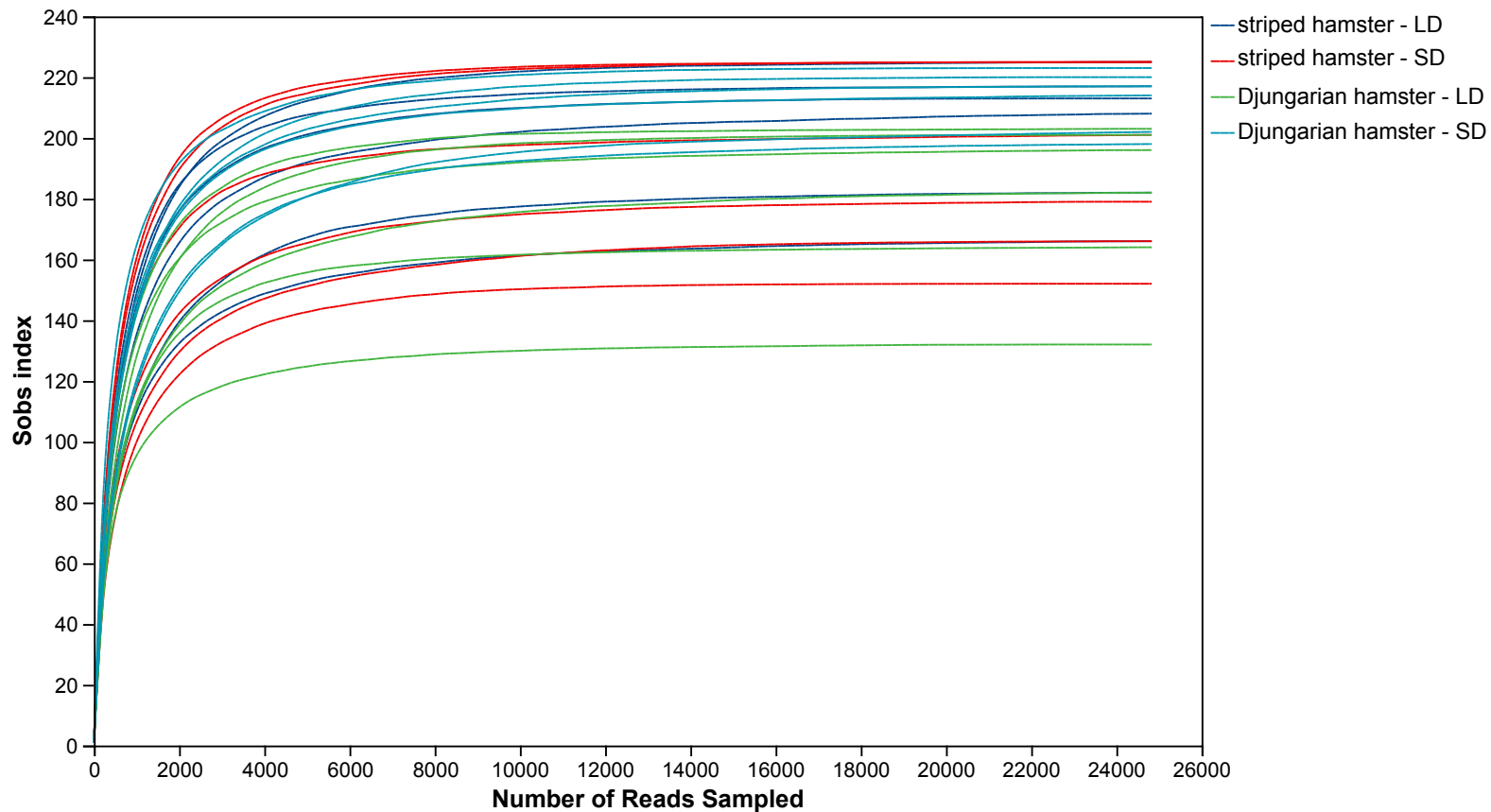

Supplement: Supplementary file 1 [file animals-15-01648-s001.zip › Figure S1.pdf]
